# A Few Words about Our Hospitals

**Published:** 1888-06-09

**Authors:** 


### A Few Words for our Hospitals.

By Mrs. Gladstone.

It is surely a humiliation to this great country that these "few words," or any words, should be needed to plead a cause that speaks so loudly for itself, and that appeals to "all sorts and conditions" of men, women, and children.

And yet, alas! "more money" is the pressing cry of all London hospitals.

Year after year an opportunity presents itself to all to listen to the cry; and now that "Hospital Sunday Week" is come round again, shall we not make a greater effort than ever before to meet the urgent need?

There must surely be deep ignorance in the minds of many of the public to account for the prevailing indifference on the subject. Let me, then, implore my readers to inform themselves of the facts of the case; let them think over some points which perhaps have not hitherto come before them; let them weigh the results of the noble work done daily and hourly under hospital roofs; and, as feelings are thus aroused, a sense of obligation will surely awaken also, and in doubled and trebled collections this week our hospitals will reap the benefit.

I leave to abler and more experienced writers the more important details of the subject, and will only venture upon a few words of a general nature.

And in the first place let me call attention to the following facts and figures of the current year, which are taken from the Special Hospital Sunday Fund number of The Hospital. The total money value represented by the actual expenditure on the relief afforded by the Hospitals and Dispensaries of the metropolis in 1887 was £651,802, or something like ten shillings and fivepence per person relieved. Towards defraying the cost of this relief, the charitable and proprietary revenue, with the patients' payments, proved inadequate to the extent of £100,000, a deficiency which would have been half as much again but for the money subscribed on Hospital Sunday, 1887. Only 6,727 beds out of 9,372 available were, however, constantly occupied, or the actual deficiency would have amounted to quite £200,000. There is only space to give the totals here, but anyone interested will find full particulars of each and all the institutions in the paper from which these figures are taken. Enough has been said, however, to show that at least £100,000 is needed on Hospital Sunday, June 10th inst.

A certain prejudice is apt to prevail against hospitals on the score that they form a kind of "happy hunting-ground" for the training of doctors and surgeons. Now it would be foolish to deny that careful and conscientious supervision must always be necessary to prevent rash, or cruel, or otherwise objectionable experiments on poor afflicted human beings. But people are inclined to forget that the training of doctors and surgeons by means of hospital practice is not a mere selfish indulgence in the interests of the students, but is really in the interests of suffering humanity. The experience, the skill, the manual dexterity, the close observation, the varied resources acquired—and only acquired—in hospital practice, are surely of incalculable importance in this world of pain and sickness. Neither must we forget that the last thirty years have (we might almost say) created the noble and devoted band of nurses trained in hospitals, and bringing to their work of mercy all the light and blessing and comfort of a religious spirit and a loving heart, coupled with the skill which comes of constant and varied experience.

Those who have visited the sick beds of a hospital ward know what tender care, what ability, what untiring kindness are bestowed upon both body and soul by nurses, physicians, surgeons, chaplains. How manifold are the comforts and advantages by which the sick are spared all but the inevitable suffering of their lot! And when visitors notice, as they cannot fail to do, the pervading patience and gentleness of tone among the patients themselves, an endurance of suffering among poor rough ignorant creatures that calls forth thankful admiration, they must not forget how much is due to the surrounding influences of the hospital. Different, indeed, would be the scene were the patients to lie sick in their small, crowded, ill-ventilated homes, devoid of all helps and comforts.

The crying need of funds is the more provoking (if I may use the word) when, thank God, workers abound. It is bad enough to have fields white unto harvest, and no labourers to reap them; it is almost worse to have the labourers waiting, sickle in hand, and no money to set them to work. Self-denying and well-qualified people are in the midst of us, who go to the root of the matter. "Silver and gold have they none," but they give their time, their work of head, hands, and heart, and too often their health. On the other side stand the multitudes who cannot, or will not, from various circumstances, thus sacrifice themselves; surely it is a small thing to ask them to help in another way. I have been met, when pleading for money for a hospital, by the pleasant answer: "Yes, indeed, you shall have my subscription; it is easy to give money; those who really deserve thanks are the workers who spend themselves in these deeds of mercy."

Times are hard it is true, and appeals are many. I do not ask my readers to give of their superfluity, for then I should ask of a very limited public. I implore those who have no "superfluity" to see what self-denial can do: what a good hard push of many shoulders to the wheel can bring about.

And I do not apologise for concluding my "few words" with a hackneyed quotation, inasmuch as the well-known lines convey so beautifully their inspiring lesson:

"The quality of mercy is not strained;
It droppeth as the gentle dew from heaven.
. . . it is twice blessed,—
It blesseth him that gives and him that takes."

(Signed)

Catherine Gladstone.

